# No sex-specific effects of balance training on dynamic balance performance in healthy children

**DOI:** 10.3389/fspor.2022.1019093

**Published:** 2022-10-18

**Authors:** Thomas Muehlbauer, Simon Schedler

**Affiliations:** Division of Movement and Training Sciences/Biomechanics of Sport, University of Duisburg-Essen, Essen, Germany

**Keywords:** postural control, childhood, pediatric exercise, trainability, maturation

## Abstract

**Background:**

Cross-sectional studies in children reported better balance performance for girls than for boys. Thus, balance trainability might be different between female and male children. The aim of the present study was to examine the effects of balance training (BT) on dynamic balance performance in girls compared to boys.

**Methods:**

Seventeen girls (age: 11.1 ± 0.7 years) and 22 boys (age: 11.1 ± 0.8 years) were assigned to either a BT-group or an active control (CON) group. BT was conducted over eight weeks (two sessions/week) while the CON-groups received their regular physical education lessons during the same period. Before and after treatment, dynamic balance performance was assessed by using the Lower Quarter Y-Balance (YBT-LQ) test. A series of three-way analyses of covariance (ANCOVA) were undertaken to test for within-between effects of Test [×2 (pretest vs. posttest)], Group [×2 (BT-group vs. CON-group)] and Sex [×2 (boys vs. girls)].

**Results:**

The three-way ANCOVA yielded a significant main effect of Test (*p* = 0.002–0.043, $\eta_{\text{p}}^{2}$ = 0.122–0.262) and of Group (all *p* < 0.001, $\eta_{\text{p}}^{2}$ = 0.330–0.651) but not of sex for all YBT-LQ reach directions and the composite score. Further, there were significant Test × Group interactions (all *p* < 0.001, $\eta_{\text{p}}^{2}$ = 0.330–0.651) in favor of both BT-groups but neither Test × Sex nor Test × Group × Sex interactions were detected.

**Conclusions:**

We conclude that BT is an effective treatment to improve dynamic balance performance in healthy children regardless of their sex. Consequently, girls and boys can be provided with the same BT regime to enhance their postural control.

## Introduction

Several studies investigated sex differences in balance performance in children and showed better values in girls compared to boys for measures of static and dynamic balance performance (1–3). For example, Nolan et al. (2) examined balance in children and reported less sway velocity and shorter sway distance in girls than in boys at 9–10 years of age for the bipedal stance with eyes open and eyes closed. Further, Steindl et al. (1) applied the Sensory Organization Test to children and showed that girls outperformed boys until the age of twelve years. Lastly, Thevenon et al. (3) asked children (age range: 6–12 years) to walk at their self-selected speed over a 7-m distance and found predominantly faster gait velocities in girls compared to same-aged boys. The aforementioned discrepancies in balance performance in favor of girls have especially been attributed to the advanced maturational level of the postural control system in girls (4, 5). Consequently, the trainability of balance might also be different in female compared to male children, which would be of major importance for practitioners (e.g., sports coaches, P.E. teachers) who develop and/or conduct balance training (BT) programs for/with mixed-sex groups.

In this regard, a systematic review with meta-analysis by Gebel et al. (6) showed a larger effectiveness of BT on balance performance for boys (effect size: 1.07) than for girls (effect size: 0.42). However, this finding resulted from an indirect comparison of different studies in which either girls or boys were examined. Therefore, it remains unclear whether boys are actually more trainable than girls or whether this finding is confounded by differences in the applied methodology (i.e., modalities of training and testing) across studies. A direct comparison of girls and boys regarding the promotion of balance performance has only been carried out in one study so far. Specifically, Schedler et al. (7) compared the learning of a balance task (i.e., balancing on a stabilometer) between female and male children (mean age: 8.5 ± 0.5 years). Across 2 days of practice, they reported balance improvements for both sexes, but the effect of learning tested on the third day was significantly larger for girls than for boys in the retention as well as the transfer test. However, transferability of the findings on balance practice to BT is questionable, since only one task (stabilometer) was practiced over a relatively short period of time (2 days of practice) with a participant-to-examiner ratio of 1:1 in a laboratory setting. In contrast, BT is characterized by performing a variety of exercises (i.e., multiple stance and walking conditions) over several weeks as a group under field conditions.

Therefore, the goal of the present study was to compare the effects of BT on dynamic balance performance between female and male children. Based on the reports (1–3) of better balance performance in girls compared to boys and the findings from Schedler et al. (7), it was hypothesized that girls compared to boys will show a greater trainability of balance.

## Materials and methods

### Participants

Thirty-nine, healthy-weight (10th percentile <BMI <90th percentile) children (17 girls, 22 boys) from two secondary school classes participated in this study after experimental procedures were explained. The classes were randomly assigned to either a BT-group or a control (CON) group. None of the participants had any history of diagnosed intellectual disabilities and/or musculoskeletal or neurological disorders that might have affected their ability to execute the BT lessons, the physical education (P.E.) lessons, or the balance assessment. Table 1 shows the characteristics of the study participants per group. Maturity offset was calculated in terms of years from peak height velocity (PHV) for each participant by using sex-specific equations provided by Moore et al. (8). Negative values indicate that the growth spurt has not yet been reached, whereas positive values illustrate that individuals have already passed their maximal growth rate. Before the start of the study, participants' assent and parents' written informed consent was obtained. The study protocol was approved by the local ethics committee (approval number: TM_10.07.17).

**Table 1:** Characteristics of the study participants (*N* = 39).

**Characteristics**	**Girls (*****n*** = **17)**		**Boys (*****n*** = **22)**	
	**BT-group (n = 8)**	**CON-group (*n* = 9)**	**BT-group (n = 12)**	**CON-group (n = 10)**
Age (years)	10.5 ± 0.5	11.6 ± 0.5	10.6 ± 0.5	11.8 ± 0.4
Maturity offset^a^ (years from PHV)	−0.91 ± 0.40	−0.18 ± 0.56	−2.21 ± 0.49	−1.41 ± 0.52
Body height (cm)	153.4 ± 3.4	154.1 ± 6.6	151.2 ± 7.9	154.4 ± 8.5
Body mass (kg)	40.8 ± 4.3	49.3 ± 11.8	40.6 ± 6.4	46.2 ± 10.4
BMI (kg/m^2^)	17.3 ± 1.6	20.5 ± 3.6	17.7 ± 2.4	19.4 ± 2.9
Leg length (cm)	92.8 ± 4.8	91.9 ± 6.5	91.7 ± 6.9	90.7 ± 4.1
Leg dominance (l, r)	2/6	0/9	1/11	2/8

Values are group means ± standard deviations.

^a^The maturity offset was calculated by using the formula provided by Moore et al. (8). Post-hoc comparisons indicate that the participants in the CON-groups were significantly older than those in the corresponding BT-groups and that the girls were more mature than the boys.

BMI, Body-Mass-Index; BT, balance training group; CON, active control group (i.e., regular physical education); l, left; r, right; PHV, peak height velocity.

### Assessment of dynamic balance performance

Dynamic balance performance was assessed during regular P.E. lessons using the Lower Quarter Y-Balance (YBT-LQ) test, which has shown excellent test-retest reliability (ICCs > 0.75) in the investigated age group (9). The participants were asked to stand on the centralized stance platform of the Y Balance Test Kit (Functional Movement Systems^®^, Chatham, USA) to which three pipes were attached that represent the anterior (AT), posteromedial (PM), and posterolateral (PL) reach directions and instructed to move a reach indicator along the pipes with one leg as far as possible. Due to limited time during the assessments only the dominant leg (i.e., kicking leg as stated by self-report) was used for reaching. In accordance to the recommendations of Plisky (10), each participant performed three practice trials followed by three data-collection trials for each reach direction from which the trial with the greatest reach distance per direction (cm) was used for subsequent analysis. The maximal reach distance per direction was normalized to leg length (LL in cm) and expressed as percentage value (%LL). In addition, the normalized composite score (CS) was computed as the sum of the three maximal absolute reach distances (cm) divided by three times LL (cm) and then multiplied by 100 and used for analysis as well. Length of the leg was measured from the anterior superior iliac spine to the most distal portion of the medial malleolus using an anthropometric measuring tape.

### Treatments

For both groups the respective treatment took place over 8 weeks (2 sessions per week, 60 mins each) at the school gym and was supervised by the P.E. teacher. Each session started with a 10- to 15-mins warm-up and finished with a 5- to 10-mins cool-down. In between, participants in the BT-group conducted static (e.g., bipedal/tandem/unipedal stance), dynamic (e.g., balancing for-/back-/sideward), proactive (e.g., weight shifting for-/back-/sideward and reaching for-/back-/sideward with one leg/arm), and reactive (e.g., push/pull while standing/walking) balance exercises. Per exercise, four sets of 30–60 s per set were performed. The rest period between sets and exercises amounted to 30 and 60 s, respectively. Progression of BT was achieved by a reduction of the support base (i.e., from bipedal over tandem to unipedal stance), a removal of visual stimuli (i.e., eyes closed), and a manipulation of proprioceptive information (i.e., use of soft mats, ankle discs, and air cushions). The participants in the CON-group underwent their regular P.E. lessons including gymnastics in the first 4 weeks and swimming in the last 4 weeks.

### Statistical analyses

An *a priori* power analysis using G ^*^ Power, version 3.1.9.2 (11) (assuming *f* = 0.25, α = 0.05, 1–β = 0.80, *r* = 0.70, 4 groups, 2 measurements, drop-out rate of 20% due to injury reasons not attributable to treatment) showed that a minimum of 38 participants would be required to be able to detect a significant medium-sized test × group × sex interaction.

Assumptions of normality (Shapiro–Wilk Test) and homogeneity of variance/sphericity (Mauchly Test) were checked and met prior to conducting further statistical analyses. Descriptive data were presented as group mean values ± standard deviations. Afterwards, an analysis of variance (ANOVA) was conducted to test for significant differences in participants characteristics and pretest values between the groups. Significant group differences occurred for all YBT-LQ reach directions and the composite score as well as for body mass and maturity offset and where thus included as covariates in the statistical analyses. Thereafter, separate (per outcome measures) 2 (Test: pretest, posttest) × 2 (Group: BT-group, CON-group) × 2 (Sex: boys, girls) analyses of covariance (ANCOVA) with repeated measures on Test were performed. If significant interactions occurred, *post-hoc* analyses (i.e., paired *t*-tests) using Bonferroni-adjusted α were performed. Lastly, effect sizes were calculated, reported as partial eta-squared ($\eta_{\text{p}}^{2}$) and classified as small (0.02 ≤ $\eta_{\text{p}}^{2}$ ≤ 0.12), medium (0.13 ≤ $\eta_{\text{p}}^{2}$ ≤ 0.25), and large ($\eta_{\text{p}}^{2}$ ≥ 0.26) (12). All statistical analyses were performed using Statistical Package for Social Sciences, version 27.0. The significance level was set at *p* < 0.05.

## Results

**Figure 1:**
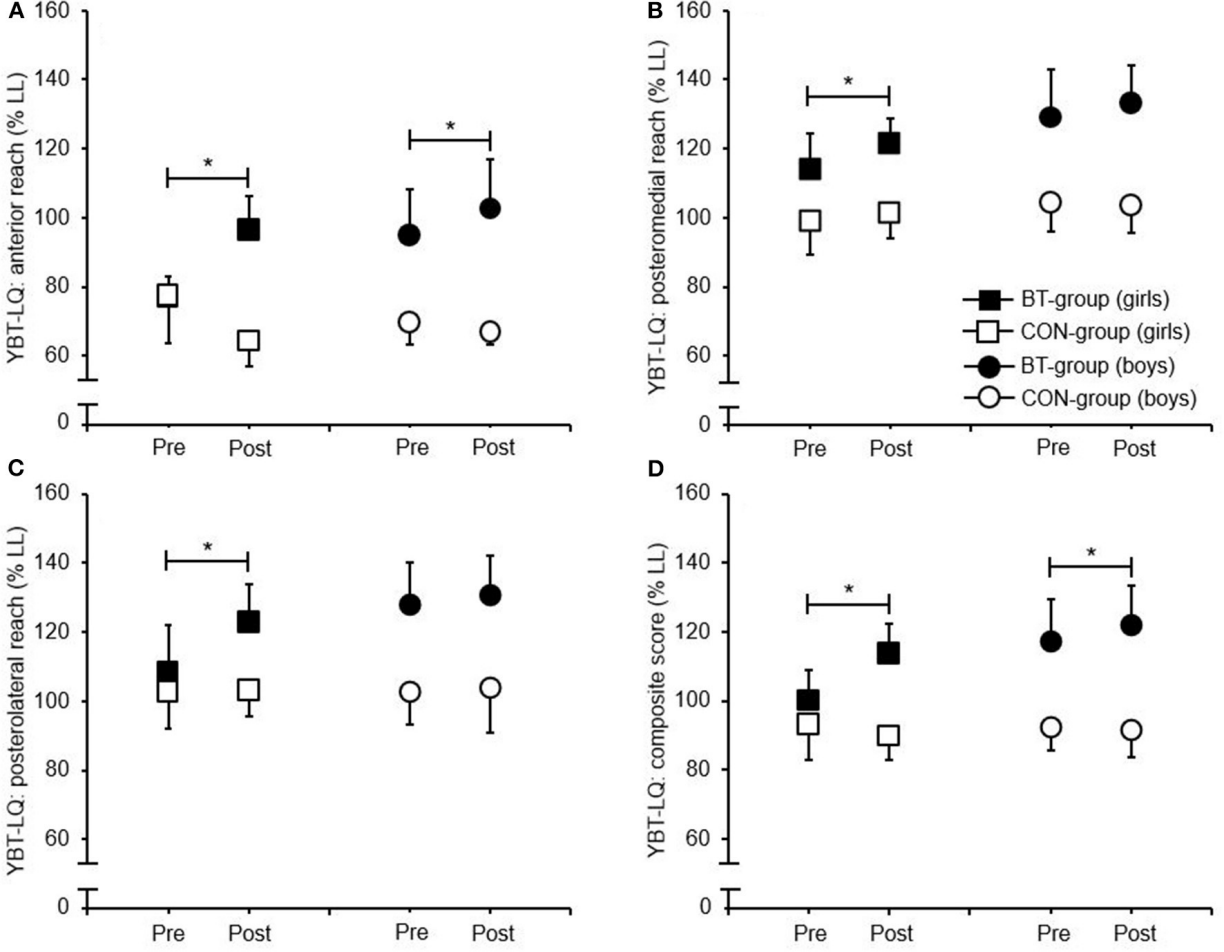
Group-specific performance changes (mean ± standard deviation) from the pretest to the posttest for the Lower Quarter Y-Balance test [**(A)** anterior reach distance; **(B)** posteromedial reach distance; **(C)** posterolateral reach distance; **(D)** composite score]. *Represents a statistically significant performance enhancement from the pretest to the posttest in favor of the BT-group (*p* < 0.05). BT, balance training group; CON, active control group (i.e., received regular physical education lessons); LL, leg length; YBT-LQ, lower quarter Y-balance test.

All participants received their treatment (i.e., BT or P.E. lessons) as initially allocated. None of the participants reported any test- or training-related injury. Descriptive and inference statistics for all analyzed variables are shown in Table 2 and Table 3 respectively. The three-way ANCOVA revealed a significant main effect of Test (*p* = 0.002–0.043, $\eta_{\text{p}}^{2}$ = 0.122–0.262) and of Group (all *p* < 0.001, $\eta_{\text{p}}^{2}$ = 0.330–0.651) but not of Sex for all YBT-LQ reach directions and the CS. Further, there were no Test × Sex interactions but significant Test × Group interactions (all *p* < 0.001, $\eta_{\text{p}}^{2}$ = 0.330–0.651). *Post-hoc* analyses yielded significant performance enhancements from the pretest to the post-test in favor of both BT-groups. Precisely, the female BT-group showed improvements for all YBT-LQ reach directions (AT: *p* < 0.001, $\eta_{\text{p}}^{2}$ = 0.507, PM: *p* = 0.022, $\eta_{\text{p}}^{2}$ = 0.142, PL: *p* = 0.001, $\eta_{\text{p}}^{2}$ = 0.222) and the CS (*p* < 0.001, $\eta_{\text{p}}^{2}$ = 0.366) and the male BT-group for the AT reach direction (*p* = 0.022, $\eta_{\text{p}}^{2}$ = 0.077) and the CS (*p* = 0.038, $\eta_{\text{p}}^{2}$ = 0.045) only (Figures 1A–D). In addition, no Test × Group × Sex interactions were detected.

**Table 2:** Performance changes from the pretest to the posttest per outcome measure.

**Outcome**	**Girls (*****n*** = **17)**				**Boys (*****n*** = **22)**			
	**BT-group (n** = **8)**		**CON-group (n** = **9)**		**BT-group (n** = **12)**		**CON-group (n** = **10)**	
	**Pretest**	**Posttest**	**Pretest**	**Posttest**	**Pretest**	**Posttest**	**Pretest**	**Posttest**
AT (%LL)	77.0 ± 5.8	96.2 ± 9.9*	77.5 ± 13.7	64.3 ± 7.3*	94.6 ± 13.6	102.6 ± 14.5*	69.7 ± 6.4	66.8 ± 6.6
PM (%LL)	114.5 ± 9.8	121.8 ± 6.9*	99.1 ± 10.0	101.7 ± 7.8	128.9 ± 14.1	133.2 ± 10.9*	104.2 ± 8.2	103.4 ± 7.9
PL (%LL)	108.2 ± 14.0	122.7 ± 11.0*	102.5 ± 10.4	103.0 ± 7.3	127.8 ± 12.4	130.9 ± 11.1*	102.9 ± 9.8	104.2 ± 13.5
CS (%LL)	99.9 ± 9.1	113.6 ± 8.8*	93.0 ± 10.2	89.7 ± 6.6	117.1 ± 12.3	122.2 ± 11.1*	92.3 ± 6.7	91.5 ± 7.7

Values are means ± standard deviations.

*Represents a statistically significant difference to the pretest value (p < 0.05).

AT, anterior reach distance; BT, balance training group; CON, active control group (i.e., regular physical education); CS, composite score; LL, leg length; PL, posterolateral reach distance; PM, posteromedial reach distance.

**Table 3:** Main and interaction effects of the repeated measures ANCOVA^*^ per outcome measure.

**Outcome**	**Main effect: Test**	**Main effect: Group**	**Main effect: Sex**	**Interaction effect: Test × Group**	**Interaction effect: Test × Sex**	**Interaction effect: Test × Group × Sex**
AT (%LL)	0.009 (0.194)	<0.001 (0.651)	0.475 (0.016)	<0.001 (0.651)	0.475 (0.016)	0.172 (0.058)
PM (%LL)	0.002 (0.262)	<0.001 (0.353)	0.976 (0.001)	<0.001 (0.353)	0.976 (0.001)	0.265 (0.039)
PL (%LL)	0.004 (0.234)	<0.001 (0.330)	0.852 (0.001)	<0.001 (0.330)	0.852 (0.001)	0.501 (0.014)
CS (%LL)	0.043 (0.122)	<0.001 (0.509)	0.712 (0.004)	<0.001 (0.565)	0.634 (0.007)	0.074 (0.096)

*Due to significant baseline differences, the reaching values at the pretest, body mass, and maturity offset were included as covariates in the statistical model.

p-values and effect sizes in brackets with 0.02 ≤ $\eta_{\text{p}}^{2}$ ≤ 0.12 indicating small, 0.13 ≤ $\eta_{\text{p}}^{2}$ ≤ 0.25 medium and $\eta_{\text{p}}^{2}$ ≥ 0.26 large effects.

AT, anterior reach distance; CS, composite score; LL, leg length; PL, posterolateral reach distance; PM, posteromedial reach distance.

## Discussion

We investigated sex-related effects of BT on dynamic balance performance in healthy 11-year-old children. The main findings of the study were (1) that participants conducting BT significantly improved their balance performance compared to those in the CON-groups (i.e., received regular P.E. lessons) and (2) that BT-related enhancements in the investigated measures of dynamic balance performance were not significantly different between girls and boys.

The detected improvements in dynamic balance performance following BT are in accordance with previous studies on dynamic balance performance in children aged around 11 years (13–15). For example, Walchli et al. (14) investigated the effects of 5 weeks of child-oriented BT on unperturbed dynamic balance performance. Compared to an active control group, 12-year-olds showed significantly decreased postural sway during one-legged stance on a spinning top. Using the identical BT-program, the same authors also reported improved balance performances in 12-year-olds following anticipated as well as unanticipated perturbations (13). More precisely, after the intervention period postural sway during two-legged stance following unanticipated as well as anticipated perturbations on a free-swinging platform was significantly reduced in the BT-group compared to the CON-group. Lastly, Schedler et al. (15) compared the effects of different BT volumes on dynamic balance performance in 10–11 year-old children. Following 8 weeks of training using either a low (i.e., 4 min/session) or a high (i.e., 18–24 min/session) volume, both groups exhibited significantly increased YBT-LQ scores, whereas there were no improvements in an active control group (i.e., received regular P.E. lessons).

Contrary to our second hypothesis, BT-related performance enhancements did not show sex-specific differences. This finding is in conflict with that from a previous study (7) which investigated sex-related effects of practice on learning a balance task in healthy children. Specifically, Schedler et al. (7) compared girls and boys that practiced balancing on a stabilometer on two consecutive days. Twenty-four hours later, they detected larger learning effects for the girls than for the boys in both the retention and transfer test. However, these results were obtained in a rather artificial laboratory setting (i.e., practicing of a single, novel task for 2 days with a participant-to-examiner ratio of 1:1), whereas the present study applied BT under more realistic conditions (i.e., group training over several weeks including multiple exercises) in the field. Concerning the underlying mechanisms, it has previously been argued that differences in adaptations to BT may be explained by advanced maturation, especially of the central nervous system in girls compared to same-aged boys (4, 5). In fact, in the present study the girls in the BT-group were significantly closer to reaching their PHV (−0.91 ± 0.40 years) than the boys in the BT-group (−2.21 ± 0.49 years), indicating a maturational advantage for the girls. Thus, in order to clarify whether sex-related differences in adaptations to BT in youth actually result from maturational differences, future studies should match participants according to their biological rather than chronological age.

In sum, the aforementioned findings and the observed results of the present study indicate that BT is an effective means to improve dynamic balance performance in healthy children, with no differences between girls and boys. From a practical point of view, it can be deduced that in order to improve dynamic balance performance in healthy 11-year-olds, girls and boys can be provided with the same BT regime.

## Limitations

There are a few limitations in this study, which have to be addressed. First, we did not record regular physical activity of our subjects, which may have influenced our results. Second, BT included exercises for all types of balance (e.g., static, dynamic, proactive, reactive), whereas only dynamic balance was tested. Third, assessments of dynamic balance performance were limited to the dominant leg due to restricted time for testing during regular P.E. lessons.

## Conclusions

The present study investigated sex-related differences in the trainability of balance in healthy children. Irrespective of sex, both BT-groups improved their balance performance compared to the CON-groups that performed their regular P.E. lessons. However, there were no sex-specific differences in performance enhancements following BT. Our findings indicate that regardless of sex BT is an effective treatment to improve dynamic balance in healthy children.

## Data availability statement

The raw data supporting the conclusions of this article will be made available by the authors, without undue reservation.

## Ethics statement

The studies involving human participants were reviewed and approved by Human Ethics Committee at the University of Duisburg-Essen, Faculty of Educational Sciences. Written informed consent to participate in this study was provided by the participants' legal guardian/next of kin.

## Author contributions

SS conducted the testings and data collections. TM analyzed the data. TM and SS wrote the main parts of the manuscript. Both authors designed the research question, contributed to critical review of draft manuscripts, and approved the final manuscript.

## Funding

The support by the Open Access Publication Fund of the University of Duisburg-Essen is acknowledged. The funding body is independent of the design of the study and collection, analysis, and interpretation of data and in writing the manuscript. Open access funding enabled and organized by the project DEAL.

## Conflict of interest

The authors declare that the research was conducted in the absence of any commercial or financial relationships that could be construed as a potential conflict of interest.

## Publisher's note

All claims expressed in this article are solely those of the authors and do not necessarily represent those of their affiliated organizations, or those of the publisher, the editors and the reviewers. Any product that may be evaluated in this article, or claim that may be made by its manufacturer, is not guaranteed or endorsed by the publisher.
